# Supplementary material for: Dairy Consumption and the Colonic Mucosa-Associated Gut Microbiota in Humans—A Preliminary Investigation
Source: Nutrients. 2025 Feb 2;17(3):567. doi: 10.3390/nu17030567 (PMC11820694; doi:10.3390/nu17030567)
Supplement: Supplementary file 1 [file nutrients-17-00567-s001.zip › nutrients-3432091-supplementary.pdf]

Supplemental Table S1. Relative abundance (%) of the major bacterial phyla by dairy product intake

Type (median intake[1])	Phylum	Relative abundance (%)		*q* value[2]
		Lower Intake	Higher Intake	
**Total dairy** (0.57 milk-equivalent serving)	*Firmicutes*	44.5	49.9	0.06
	Bacteroidetes	42.6	32.9	**0.007**
	Proteobacteria	9.92	9.76	0.70
	Verrucomicrobia	1.71	3.94	**0.02**
	Fusobacteria	0.89	2.66	**0.006**
	Actinobacteria	0.29	0.68	**0.03**
**Milk** (0.24 cup)	*Firmicutes*	49.8	45.6	0.28
	Bacteroidetes	36.4	37.3	0.62
	Proteobacteria	8.79	10.69	0.08
	Verrucomicrobia	1.05	4.66	**< 0.0001**
	Fusobacteria	3.49	0.64	0.86
	Actinobacteria	0.40	0.61	0.54
**Cheese** (0.27 milk-equivalent serving)	*Firmicutes*	45.9	49.6	0.24
	Bacteroidetes	43.0	30.2	**< 0.0001**
	Proteobacteria	7.90	12.0	0.24
	Verrucomicrobia	2.25	3.88	**0.01**
	Fusobacteria	0.19	3.87	**0.0007**
	Actinobacteria	0.66	0.36	0.26
**Yogurt** (0.002 cup)	*Firmicutes*	47.6	47.7	0.91
	Bacteroidetes	41.2	32.4	**0.032**
	Proteobacteria	8.37	11.9	0.10
	Verrucomicrobia	1.73	4.40	0.91
	Fusobacteria	0.49	3.46	0.09
	Actinobacteria	0.41	0.64	0.91

[1]Higher vs. lower intake was dichotomized using the median intake of each food item in 34 participants. For yogurt, the comparison was between no yogurt and had yogurt intake.

[2]The bacteria phylum was ordered according to the relative abundance. The statistically significant *q* value was bolded.

Supplemental Table S2. Relative abundance (%) of major bacterial genera by dairy products intake[1]

Type (median intake[2])	Genus	Relative abundance (%)		q value
		Lower Intake	Higher Intake	
**Total dairy**	*Bacteroides*	36.4	24.5	**0.01**
	Faecalibacterium	5.51	11.2	**0.0007**
	Akkermansia	1.71	3.94	0.05
	Fusobacterium	0.85	2.65	**0.01**
	Lachnoclostridium	2.31	1.58	0.05
	Haemophilus	0.78	1.59	**0.01**
	Erysipelatoclostridium	1.59	1.19	0.05
	Bifidobacterium	0.05	0.25	0.05
**Milk**	*Faecalibacterium*	5.77	11.3	**0.007**
	Akkermansia	1.08	4.57	**<0.0001**
	Roseburia	1.09	3.75	**0.0003**
	Parabacteroides	0.63	2.23	**0.005**
	Haemophilus	0.55	1.81	**0.008**
	Alistipes	0.75	1.69	**0.02**
	Lachnoclostridium	2.31	1.53	**0.03**
	Bifidobacterium	0.09	0.23	0.88
**Cheese**	*Bacteroides*	33.6	29.4	**0.03**
	Parabacteroides	2.16	0.81	**0.0009**
	Escherichia/Shigella	2.65	7.58	**0.049**
	Fusobacterium	0.18	3.82	**0.004**
	Akkermansia	2.25	3.88	**0.03**
	Subdoligranulum	2.52	1.20	**<0.0001**
	Alistipes	1.72	0.78	**0.05**
	Bifidobacterium	0.23	0.10	0.15
**Yogurt**	*Lachnospiraceae (unc91005)*	8.20	5.79	0.045
	Bifidobacterium	0.11	0.22	0.15

[1]Only the major genera (> 1% relative abundance) with the statistically significant difference and *Bifidobacterium* were shown in the table.

[2]Higher vs. lower intake was dichotomized using the median intake of each food item in 34 participants. For yogurt, the comparison was between no yogurt and had yogurt intake.

Supplemental Table S3. Relative abundance of bacterial genera by dairy consumption in sigmoid samples

**Type** (median intake[‡])	**Genus**	**Relative abundance (%)**		***q* value**
		**Lower Intake**	**Higher Intake**	
Total Dairy	*Faecalibacterium*	7.36	12.5	0.02
	Bifidobacterium	0.01	0.28	0.04
Milk	*Faecalibacterium*	5.74	11.5	0.004
	Akkermansia	1.05	4.65	< 0.0001
Cheese	*Bacteroides*	35.9	24.2	0.08
	Subdoligranulum	2.52	1.20	<0.0001
